# DOT1L inhibition does not modify the sensitivity of cutaneous T cell lymphoma to pan-HDAC inhibitors *in vitro*

**DOI:** 10.3389/fgene.2022.1032958

**Published:** 2022-11-08

**Authors:** Eliza Mari Kwesi-Maliepaard, Muddassir Malik, Tibor van Welsem, Remco van Doorn, Maarten H. Vermeer, Hanneke Vlaming, Heinz Jacobs, Fred van Leeuwen

**Affiliations:** ^1^ Division of Gene Regulation, Netherlands Cancer Institute, Amsterdam, Netherlands; ^2^ Department of Dermatology, Leiden University Medical Center, Leiden, Netherlands; ^3^ Division of Tumor Biology and Immunology, Netherlands Cancer Institute, Amsterdam, Netherlands; ^4^ Department of Medical Biology, Amsterdam UMC, University of Amsterdam, Amsterdam, Netherlands

**Keywords:** DOT1L, HDAC, histone acetylation, epigenetics, cutaneous T-cell lymphoma (CTCL), histone methyl transferase (HMT), histone deacetylase inhibitors

## Abstract

Cutaneous T-cell lymphomas (CTCLs) are a subset of T-cell malignancies presenting in the skin. The treatment options for CTCL, in particular in advanced stages, are limited. One of the emerging therapies for CTCL is treatment with histone deacetylase (HDAC) inhibitors. We recently discovered an evolutionarily conserved crosstalk between HDAC1, one of the targets of HDAC inhibitors, and the histone methyltransferase DOT1L. HDAC1 negatively regulates DOT1L activity in yeast, mouse thymocytes, and mouse thymic lymphoma. Here we studied the functional relationship between HDAC inhibitors and DOT1L in two human CTCL cell lines, specifically addressing the question whether the crosstalk between DOT1L and HDAC1 observed in mouse T cells plays a role in the therapeutic effect of clinically relevant broad-acting HDAC inhibitors in the treatment of human CTCL. We confirmed that human CTCL cell lines were sensitive to treatment with pan-HDAC inhibitors. In contrast, the cell lines were not sensitive to DOT1L inhibitors. Combining both types of inhibitors did neither enhance nor suppress the inhibitory effect of HDAC inhibitors on CTCL cells. Thus our *in vitro* studies suggest that the effect of commonly used pan-HDAC inhibitors in CTCL cells relies on downstream effects other than DOT1L misregulation.

## Introduction

Cutaneous T-cell lymphoma (CTCL) is a heterogeneous group of lymphomas that manifest in the skin. The most common types are mycosis fungoides (MF) and Sézary syndrome (SS) that commonly originate from the CD4^+^ T-cell lineage (Willemze et al., 2005; García-Díaz et al., 2021). MF is characterized by patches and plaques on the skin that can progress into tumors and has an indolent clinical behavior (Willemze et al., 2005; Agar et al., 2010; García-Díaz et al., 2021). SS presents with erythroderma, lymphadenopathy and presence of malignant T cells in the blood and has a poor survival rate (Willemze et al., 2005; Agar et al., 2010; García-Díaz et al., 2021). Treatment of MF/SS depends on the disease stage, with early-stage disease mostly treated with local skin-directed therapies, whereas more advanced-stage disease is treated with systemic therapies (Whittaker et al., 2016; Wilcox, 2016; Scarisbrick et al., 2021).

One of the emerging systemic therapies that show promising results is treatment with histone deacetylase (HDAC) inhibitors (Mann et al., 2007; Jones et al., 2016; Whittaker et al., 2016; Wilcox, 2016; Lopez et al., 2018; Sermer et al., 2019; Chen et al., 2020; Lai and Wang, 2021b; Pavlidis et al., 2021; Scarisbrick et al., 2021). HDACs catalyze the removal of acetyl groups from histones, resulting in reduced histone acetylation levels, reduced chromatin accessibility and gene silencing (Clayton et al., 2006). Treatment with HDAC inhibitors results in increased histone acetylation levels and has been proposed to induce growth arrest and apoptosis (Zhang et al., 2005; Marks, 2007; Tiffon et al., 2011; West and Johnstone, 2014; Lopez et al., 2018; Lai and Wang, 2021b; Ramaiah et al., 2021). However, most commonly used HDAC inhibitors target multiple HDACs and HDACs act on a large number of nuclear and cytoplasmic proteins, extending their functions beyond histone acetylation (Falkenberg and Johnstone, 2014). Although the exact mechanism of HDAC inhibitor treatment in CTCL is not known, several HDAC inhibitors, including Vorinostat, Panobinostat and Romidepsin, have been approved or are currently in clinical trials for CTCL treatment (Mann et al., 2007; Ellis et al., 2008; Duvic et al., 2013, 2018; Barbarotta and Hurley, 2015; Jones et al., 2016; Lopez et al., 2018; Sermer et al., 2019; Chen et al., 2020; Lai and Wang, 2021b; Pavlidis et al., 2021). Vorinostat and Panobinostat are broad-acting HDAC inhibitors while Romidepsin is specific for Class I HDACs, which includes HDAC1-3 and HDAC8 (Seto and Yoshida, 2014; Shortt et al., 2017; Chen et al., 2020; Pavlidis et al., 2021).

Despite the clinical interest, the response towards HDAC inhibitors varies between patients and between different HDAC inhibitors (Fantin et al., 2008; Qu et al., 2017; Brink Buus et al., 2018; Andrews et al., 2019; Scarisbrick et al., 2021). One potential option to improve the response to HDAC inhibitors is to combine them with other (epigenetic) inhibitors (Lai and Wang, 2021a; Liu et al., 2022). Combined treatment can help to improve therapeutic outcomes and the synergy between drugs can allow for lower concentrations, resulting in a decrease in adverse events (Jones et al., 2016; Tomaselli et al., 2020). For example, combinations of HDAC inhibitors with other inhibitors, such as those targeting DNA methyltransferases and BCL2, have shown promising clinical results in CTCL, although not for all patients (Rozati et al., 2016; Kim et al., 2018; Sermer et al., 2019).

Our previous integrated genomics analysis of MF recently led to the identification of several candidate genes potentially involved in MF development. Some of the observed genomic rearrangements correspond to fusion transcripts involving genes playing a role in epigenetic regulation, such as lysine demethylase 6A (KDM6A) and DOT1-Like Histone Lysine Methyltransferase (DOT1L) (Bastidas Torres et al., 2018). We recently also discovered an evolutionarily conserved crosstalk between the class I histone deacetylase HDAC1 and DOT1L (Vlaming et al., 2019). DOT1L facilitates the methylation of lysine 79 on histone H3 (H3K79me), which is associated with active transcription (Steger et al., 2008). DOT1L inhibitors are being investigated in clinical trials for treatment of leukemia caused by rearrangement of the MLL gene (Mixed-Lineage Leukemia-rearranged) (Wang et al., 2016; Stein et al., 2018). We observed that in both yeast cells and mouse thymocytes, HDAC1 (Rpd3 in yeast) functions as a negative regulator of H3K79 methylation (Vlaming et al., 2019). Mechanistically, loss of Rpd3 in yeast leads to more histone acetylation, more transcription, more mono-ubiquitination of histone H2B, and increased transcription-associated H3K79 methylation. In the mouse, thymic lymphoma cell lines derived from conditional *Hdac1* knock-out mice also show increased H3K79me and this coincides with increased sensitivity towards DOT1L inhibitors (Vlaming et al., 2019). Cryo-electron microscopy analysis of yeast Dot1 bound to nucleosomes carrying post-translational modifications recently provided biochemical support for the cross-talk observed in cells. It appears that Dot1 uses acetylation of the tail of H4 and mono-ubiquitination of H2B to productively engage with the nucleosome surface to bind and modify H3K79, suggesting an intricate local nucleosomal signaling network around H3K79me (Valencia-Sánchez et al., 2021).

These observations lead to the question whether the observed crosstalk between DOT1L and HDAC1 in mouse T cells plays a role in the therapeutic effect of pan-HDAC inhibition in human CTCL. Predicting how HDAC1-DOT1L crosstalk affects CTCL is difficult because the role of HDACs in cancer is known to be context dependent. In mouse thymic lymphoma, HDAC1 deletion results in oncogenic transformation (Vlaming et al., 2019), whereas in the case of CTCL reduced HDAC activity has tumor-suppressing consequences (Lopez et al., 2018; Chen et al., 2020; Pavlidis et al., 2021). Context-dependent functions in tumor biology have also been described for DOT1L. For example, in MLL-rearranged leukemia DOT1L inhibition is used for treatment, whereas DOT1L ablation has also been associated with therapy resistance of mouse melanoma (Torre et al., 2021). Based on the suppressive effect of HDAC1/Rpd3 on DOT1L that we previously observed in yeast and mouse thymocytes, we here first tested the possibility that clinically relevant pan-HDAC inhibitors, which also target HDAC1, upregulate DOT1L activity to execute their therapeutic effect. In this scenario, combining HDAC inhibitor treatment with DOT1L inhibitors is expected to diminish the therapeutic effect of the pan-HDAC inhibitors. Indeed, from an epigenetic and gene-regulation perspective, HDAC inhibition is expected to lead to increased gene expression, whereas loss of DOT1L activity is expected to lead to reduced gene expression, thereby potentially leading to opposing suppressive effects if they act on the same gene targets. Based on the anti-tumor effects of DOT1L inhibitors in MLL-rearranged leukemia and thymic lymphoma, we also investigated whether DOT1L inhibitor treatment alone or in collaboration with HDAC inhibitors reduces viability of CTCL cells.

## Materials and methods

### Cell lines

The human CTCL cell line Hut-78 (ATCC number TIB-161) and SeAx are derived from the peripheral blood of patients with SS (Gootenberg et al., 1981; Kaltoft et al., 1987). The human T cell leukemia cell line Jurkat was provided by Paul van den Berk and Jannie Borst (Netherlands Cancer Institute). The MLL-rearranged lymphoma cell line MV4-11 was a gift from Maarten Fornerod (Department of Cell Biology, Erasmus Medical Center, Rotterdam, Netherlands). Hut-78 cells were cultured in DMEM media (Gibco) with 10% FBS (Sigma). SeAx and Jurkat cells were cultured in RPMI (Gibco) supplemented with 10% FBS (Sigma) and for SeAx also 200 U/ml IL-2 (Novartis). MV4-11 cells were cultured in RPMI media (Gibco) supplemented with 20% FBS (Sigma) and 5 ng/ml GM-CSF (Sigma). All media were supplemented with Penicillin/Streptomycin. Cells were cultured at 37 °C under 5% CO_2_ conditions.

### Cell viability assay

The HDAC inhibitors Panobinostat (Sigma) and Vorinostat (Sigma) and the DOT1L inhibitors Pinometostat (EPZ-5676) (Selleck Chemicals) and SGC-0946 (Selleck Chemicals), and DMSO (Sigma) were diluted in culture medium. First, the inhibitors were diluted in culture medium to a stock concentration that was twice as high as the highest concentration tested. The stock concentration was further diluted to obtain to the desired range of concentrations. Twenty-thousand cells were plated per well in a flat-bottom culture-treated 96 wells plate, in 50 µl culture medium and 50 µl culture medium with inhibitor was added. Four hours before measuring cell viability, Cell Titer Blue (Promega) was added to the wells and cells were incubated in the dark at 37°C under 5% CO_2_ conditions. After 4 hours, fluorescence was measured on the EnVision Multilabel Reader (Perkin Elmer). In order to calculate cell viability, values for fluorescence intensity of each condition were normalized against those of untreated cells. Each biological replicate represents an average of three technical replicates of an independent experiment.

### Viability by flow cytometry

Viability, early and late apoptosis was measured by Annexin V-APC (Biolegend) and DAPI (3 μg/ml; Sigma) staining. Staining was performed in 96-wells V-bottom plates and was previously optimized and titrated. Approximately 30,000–100,000 cells were washed in PBS, resuspended in 50 µL 1x binding buffer (10 mM HEPES pH 7.4, 140 mM NaCl, 2.5 mM CaCl_2_) containing 1:50 Annexin V-APC and 1:50 DAPI and subsequently incubated for 15 min at room temperature (shielded from light). After incubation the stained cells were transferred to tubes, the suspension was diluted by addition of 200 µL binding buffer and briefly kept on ice. Flow cytometry acquisition was subsequently performed using an LSR Fortessa (BD Biosciences) and the FACSDiva software v8.0.2 (BD Biosciences). Results were analyzed in FlowJo v10.5.3 (BD Biosciences).

### Western blot

Cells were seeded and cultured in 6-wells plates at two million cells (2-h incubation), one million cells (24-h incubation) and 0.5 million cells (72-h incubation) in 3 ml medium supplemented with 25 µM Pinometostat (Selleck Chemicals), 0.25 µM or 1 µM Vorinostat (Sigma), or with 0.025% DMSO (Sigma) as a vehicle control. Cells were washed with ice-cold PBS and pellets were snap-frozen in liquid nitrogen and stored at −80°C. Cells were lysed in ice cold RIPA buffer (25 mM Tris (pH 7.5), 150 mM NaCl, 1% Nonidet P-40, 1% sodium deoxycholate, 1 mM EDTA, 0.1% SDS) supplemented with complete protease inhibitor (Roche) and 5 times sheared over a 25G needle (BD). Subsequently, samples were sonicated in Eppendorf tubes for 5 min with a 30 s interval (30 s on, 30 s off) using a Bioruptor Pico sonicator (Diagenode). Lysates were centrifuged for 2 min at 16,000x g to remove cellular debris. The supernatant was stored for use at −20°C for later use. The protein concentration of the lysates was determined using the DC protein assay (BioRad). Samples were boiled at 95°C for 5 min in 5x SDS-sample buffer (250 mM Tris-HCl pH 6.8, 10% SDS, 50% glycerol, 0.5 M DTT, 0.5% bromophenol blue) and separated on 16% polyacrylamide gels or NuPAGE 4–12% Bis-Tris gels (Invitrogen). Separated proteins were transferred on a 0.2 μm nitrocellulose membrane for 1 h at 1A (histone blots) or a 0.45 μm nitrocellulose membrane for 4 h at 1 A or overnight at 0.2 A (DOT1L blots). Membranes were blocked using 5% Nutrilon (Nutricia) in PBS for 30 min. Subsequently, membranes were incubated overnight at 4°C with the primary antibodies diluted in Tris-buffered saline containing 0.05% Tween-20 (TBST) and 2% Nutrilon: anti-H3K79me1 (RRID: AB_2631105) (1:1000), anti-H3K79me2 (04-835, Millipore) (1:1000), anti-H3K9ac (ab4441, Abcam) (1:2500), anti-H3K9ac (13-0020, Epicypher) (1:1000), anti-H4Ac pan (06-866, Millipore) (1:2000), anti-H2BK120ub1 (5546, Cell Signaling Technology) (1:1000) DOT1L (90878, Cell Signaling Technology) (1:500), anti-α-Tubulin (9023948, Santa Cruz Biotechnology) (1:10000) or anti-H3 (ab1791; Abcam) (1:5000). After incubation, the membranes were washed three times with TBST and incubated with secondary antibody for 45 min in TBST with 2% Nutrilon. Secondary antibodies used (1:10,000) were IRDye 800CW goat anti-Mouse IgG (0.5 mg) 926-32210 Li-COR (RRID:AB_621842), IRDye 800CW goat anti-Rabbit IgG (0.5 mg) 926-32211 Li-COR (RRID:AB_621843), IRDye 680RD goat anti-Mouse IgG (0.5 mg) 925-68070 Li-COR (RRID:AB_2651128), IRDye 680RD goat anti-Rabbit IgG (0.5 mg) 925-68071 Li-COR (RRID AB_2721181). Finally, the membranes were washed three times with TBST and once in PBS for subsequent scanning using a LI-COR Odyssey IR Imager (LI-COR Biosciences). Some of the blots were stripped and reprobed. Antibody stripping was performed by 15 min incubation in MiliQ, followed by 3 h incubation in 0.2 M NaOH and 15 min incubation in MiliQ. Stripping was confirmed by absence of signal on the scans after incubation with secondary antibody only. Stripped blots were subsequently stained with primary and secondary antibodies as described above.

**FIGURE 1:**
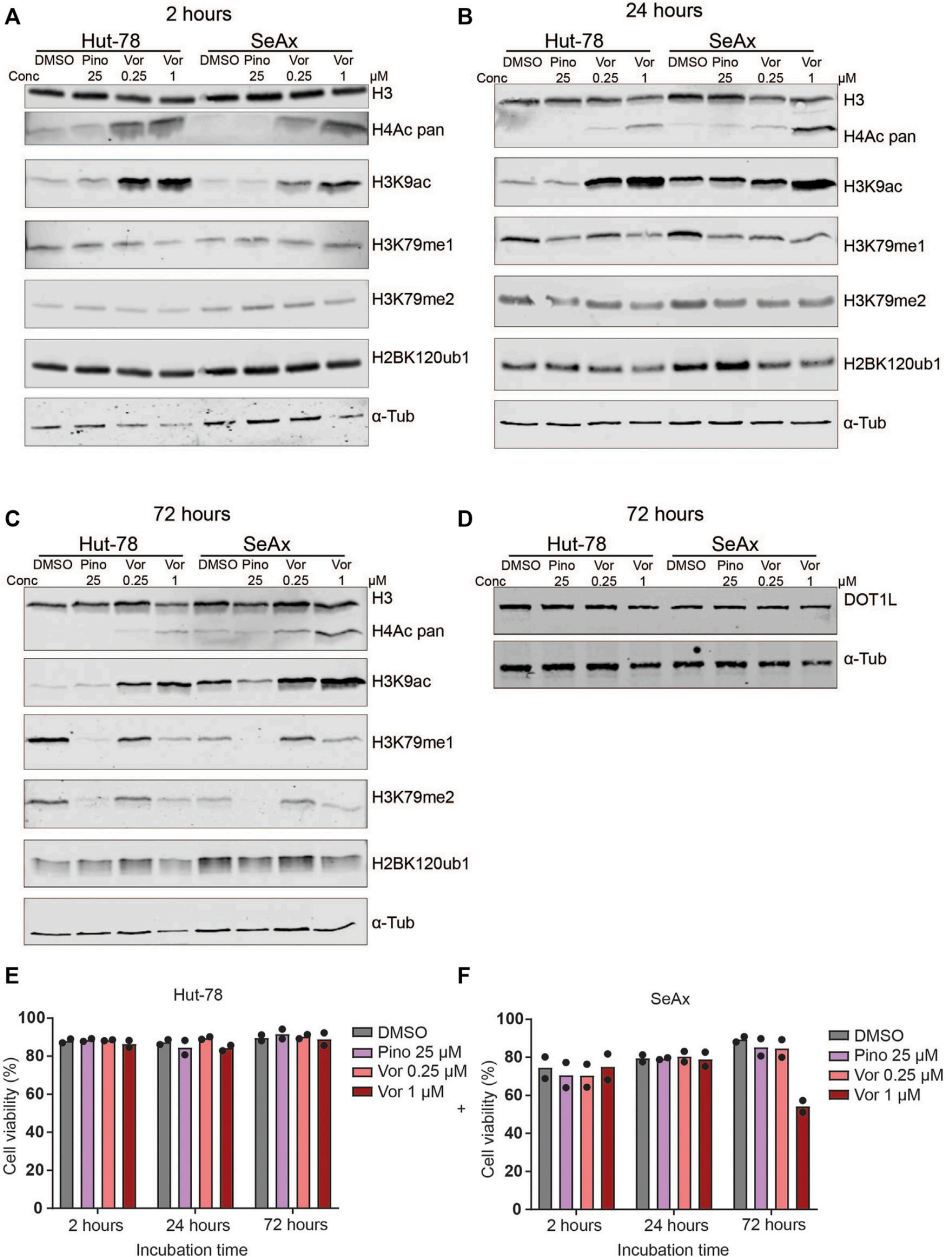
Histone modification levels after treatment of CTCL lines with HDAC inhibitors and DOT1L inhibitors. **(A–C)** Western blots showing H3K79me1/2, pan H4ac, H3K9ac and H2BK120ub1 levels in the indicated CTCL cell lines after treatment with 25 µM Pinometostat (Pino), and 0.25 µM or 1 µM Vorinostat (Vor) for 2 h **(A)**, 24 h **(B)** or 72 h **(C)**. **(D)** Western blot on unsonicated samples showing DOT1L protein levels in the indicated CTCL cell lines after treatment with 25 µM Pinometostat (Pino), 0.25 µM or 1 µM Vorinostat for 72 h. **(E–F)** Cell viability of the indicated CTCL cell lines after treatment with 25 µM Pinometostat (Pino), and 0.25 µM or 1 µM Vorinostat for 72 h determined by Annexin V-DAPI staining. Bars indicate average value of two independent biological replicates; individual data points are shown.

For the western blots in Supplementary Figure S1D–E the following protocol was used. Cells were washed with ice-cold PBS supplemented with PMSF (Sigma) and 5 mM sodium butyrate (Sigma) or 5 µM Trichostatin A (TSA) (Sigma) and pellets were frozen. Protein lysate preparation and western blotting was done as described in (Vlaming et al., 2019). Briefly, nuclear extracts were prepared by incubating cells in hypotonic lysis buffer (HLB) (10 mM Tris (pH 7.8), 5 mM MgCl2, 10 mM KCl, 0.1 mM EDTA, 300 mM sucrose) for 10 min. To rupture the cells Nonidet P-40 was added to an end concentration of 0.12% and nuclei were collected by centrifugation. Nuclei were further lysed in RIPA buffer (20 mM Tris (pH 7.5), 150 mM NaCl, 1% Nonidet P-40, 0.5% sodium deoxycholate, 1 mM EDTA, 0.1% SDS) for 30 min. All buffers were supplemented with complete protease inhibitor (Roche) and 5 mM sodium butyrate (Sigma) or 5 µM TSA (Sigma). Subsequently, samples were sonicated for 2.5 min with a 10 s interval. After this step the samples were boiled in 5x SDS-sample buffer and processed similar to the samples used in Figure 1 and Supplementary Figure S1A–C, except for a different anti-H3K79me2 antibody (RRID: AB_2631106). Western blot signals were quantified using the Image Studio software Version 5.2.5 (LI-COR) by drawing rectangles around the bands of interest, applying the median background correction method and a border width of 3 (top/bottom). Signals for each blot were normalized to untreated (DMSO) Hut-78 cells.

### Statistics

All statistical analyses were done using GraphPad Prism (version 9.4.1). Cell viability curves and half maximal effective concentration (EC50) values were determined using the ‘log (against) vs response–Find ECanything’ function, with top constraint to one and bottom constraint to 0, interpolation confidence interval: 95%, and untreated condition was set at -13 (Log10) in order to fit the line. Unpaired student’s t-test was used to determine difference between two different groups.

## Results

To address the role of DOT1L in the therapeutic effect of HDAC inhibitors, we here studied single and combination treatments of two CTCL cell lines with the HDAC Class I, II and IV inhibitors Vorinostat (SAHA) and Panobinostat, both of which show promising results in CTCL treatment (Mann et al., 2007; Ellis et al., 2008; Duvic et al., 2013; Barbarotta and Hurley, 2015; Jones et al., 2016; Lopez et al., 2018; Sermer et al., 2019; Chen et al., 2020; Lai and Wang, 2021b; Pavlidis et al., 2021), and the highly specific and clinically relevant DOT1L inhibitors Pinometostat and SGC-0946.

We first determined the effect of HDAC inhibitors on global levels of H3K79me. Cells were treated with two different concentrations of Vorinostat and H3K79me1/2 and H3K9ac and H4ac levels were assessed at different time points. Treatment with this HDAC inhibitor showed an increase in H3K9ac and H4 acetylation, serving as a proxy for global histone acetylation, confirming the reduction in HDAC activity (Figures 1A–C and Supplementary Figures S1A–E). This effect was already seen after 2 h of treatment (Figure 1A and Supplementary Figure S1A). However, no consistent differences were observed in H3K79me1 and H3K79me2 between untreated and Vorinostat-treated cells (Figures 1A–C and Supplementary Figures S1A–E, G–H). Upon treatment with the DOT1L inhibitor Pinometostat, H3K79me levels were strongly reduced after 72 h (Figure 1C and Supplementary Figures S1C, E). As expected for a histone methylation of which the loss depends on histone turnover or histone dilution due to replication (De Vos et al., 2011; Chory et al., 2019; Kwesi-Maliepaard et al., 2020), shorter treatment (2 and 24 h) resulted in no or a small reduction in H3K79me (Figures 1A,B and Supplementary Figures S1A, B, D). Although H3K79me levels were reduced after 72 h of treatment with Pinometostat, DOT1L protein levels were not affected (Figure 1D and Supplementary Figure S1F). This suggests that the DOT1L-inhibitor reduces the activity of DOT1L without affecting its stability.

To further investigate possible downstream effects of HDAC inhibitors that might affect DOT1L activity in CTCL cells, we looked at the best-known upstream regulator of H3K79 methylation. The activity of DOT1L towards H3K79 on the surface of the nucleosome is known to be enhanced by mono-ubiquitination of histone H2BK120 (H2BK120ub1) (Anderson et al., 2019; Valencia-Sánchez et al., 2019; Worden et al., 2019; Worden and Wolberger, 2019; Yao et al., 2019). Our previous studies in yeast and mouse thymocytes suggest that the histone deacetylase HDAC1/Rpd3 might, at least in part, negatively regulate DOT1L activity towards H3K79 by negative regulation of H2BK120ub1 (Vlaming et al., 2019). In this study, treatment of CTCL cell lines with the HDAC-inhibitor Vorinostat did not have a consistent effect on H2BK120ub1 (Figures 1A–C and Supplementary Figures S1A, C, I). Of note, cell viability decreased and apoptosis increased in SeAx cells treated with the highest concentration (1 µM) Vorinostat for 72 h (Figures 1E, F and Supplementary Figure S1J), leading to lower levels of protein in these samples. Taken together, based on global H3K79me levels, we could not detect an effect of HDAC inhibitors on DOT1L activity or the nucleosomal crosstalk signal that regulates it. This is in contrast to the increase in H3K79me in mouse thymocytes lacking HDAC1 (Vlaming et al., 2019), one of the targets of Vorinostat.

**FIGURE 2:**
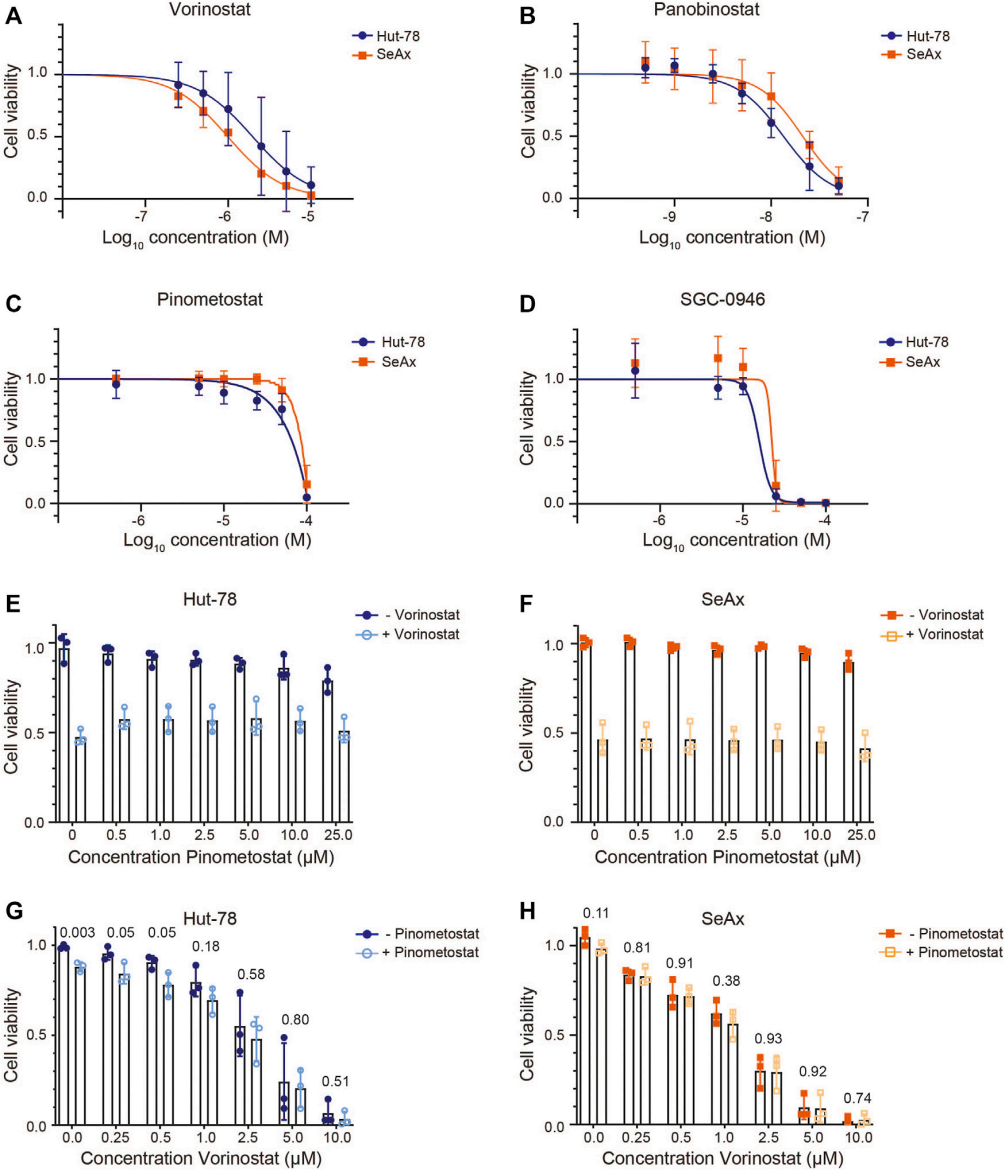
CTCL cell viability upon treatment with HDAC inhibitors and/or DOT1L inhibitors. **(A–D)** Cell viability of the CTCL cell lines Hut-78 and SeAx after 72 h of treatment with HDAC inhibitors Vorinostat **(A)** and Panobinostat **(B)** and DOT1L inhibitors Pinometostat **(C)** and SGC-0946 **(D)**. Points show average values of three independent biological replicates ±SD. **(E–F)** Cell viability of Hut-78 cells **(E)** and SeAx cells **(F)** treated with increasing concentrations of Pinometostat and with 2 µM Vorinostat (Hut-78) or 1 µM Vorinostat (SeAx) or without Vorinostat. Bar plots show average values of three independent biological replicates ±SD and individual data points. **(G–H)** Cell viability of Hut-78 **(G)** and SeAx **(H)** cells treated with increasing concentrations of Vorinostat and with or without 25 µM Pinometostat. Bar plots show average values of three independent biological replicates ±SD and individual data points. *p*-values derived from unpaired student’s t-test are indicated. Cell viability was determined using a Cell Titer Blue assay and normalized against untreated cells. Each biological replicate represents the average of three technical replicates.

We next assessed the sensitivity of the CTCL cell lines to HDAC inhibitors and DOT1L inhibitors. As expected, CTCL cell lines showed a dose-dependent sensitivity for HDAC inhibitors (Figures 2A, B). The EC_50_ levels obtained (Table 1) are in agreement with most EC_50_ levels found in other studies (Fantin et al., 2008; Heider et al., 2009; Chakraborty et al., 2013; Netchiporouk et al., 2017; Schcolnik-Cabrera et al., 2018; Li et al., 2019; Jimura et al., 2021). In order to account for any effects of the DMSO that is used to dissolve the inhibitors (maximum 0.2% DMSO), we tested cell viability of cells treated with or without DMSO. Treatment with 0.2% DMSO did not affect cell viability of the CTCL cell lines, suggesting that the cell viability effects were caused by the inhibitors (Supplementary Figure S2A). To confirm that the DOT1L inhibitors used were active, we analyzed a known DOT1L-dependent MLL-rearranged leukemia cell line (MV4-11) and a known DOT1L inhibitor insensitive T-cell leukemia cell line (Jurkat) (Daigle et al., 2013). DOT1L inhibitors showed a dose-dependent inhibitory effect on the MV4-11 cell line, the CTCL cell lines and the Jurkat cell line were insensitive to DOT1L inhibitors (Figures 2D, E and Supplementary Figures S2B, C), confirming the biological activity of the DOT1L inhibitors used in this study.

**TABLE 1:** EC_50_ values for HDAC inhibitors.

Inhibitor	Cell line	Absolute EC_50_	95% confidence interval
Vorinostat	Hut-78	2.0 µM	1.2–3.3 µM
Vorinostat	SeAx	1.0 µM	0.9–1.1 µM
Panobinostat	Hut-78	13.4 nM	11.1–16.3 nM
Panobinostat	SeAx	21.1 nM	16.0–27.6 nM

We next tested whether DOT1L inhibition can play a role in the therapeutic effect of HDAC inhibitors on CTCL lines. We first co-treated cells with an HDAC inhibitor in a concentration that resulted in an intermediate reduction in cell viability and added increasing concentrations of DOT1L inhibitor. For this experiment the HDAC inhibitor Vorinostat, which is already approved for CTCL treatment (Mann et al., 2007), and the DOT1L inhibitor Pinometostat, which is in clinical trials for MLL-rearranged leukemia (Stein et al., 2018), were chosen. After 3 days in culture, cells treated with the HDAC inhibitors showed the expected reduced viability. However, co-treatment with increasing doses of DOT1L inhibitors did not result in increased or reduced cell viability (Figures 2E, F). We next treated cells with the DOT1L inhibitor Pinometostat at a high concentration (25 µM) at which no effects on cell viability were observed in CTCL cell lines and the DOT1L-inhibitor insensitive Jurkat cell line, and co-treated with increasing concentrations of HDAC inhibitor. Also under these conditions we did not observe a synergistic or antagonistic effect between HDAC inhibitor and DOT1L inhibitor (Figure 2G, H, Table 2). Taken together, we did not observe crosstalk between HDAC inhibitor and DOT1L inhibitor treatment in cell viability in CTCL cell lines cultured *in vitro*.

**TABLE 2:** EC_50_ values after co-treatment with HDAC and DOT1L inhibitor.

Co-treatment	Cell line	Absolute EC_50_ (µM)	95% confidence interval
Vorinostat ‒ Pinometostat	Hut-78	2.6	2.1–3.1 µM
Vorinostat + Pinometostat	Hut-78	1.8	1.4–2.3 µM
Vorinostat ‒ Pinometostat	SeAx	1.2	1.0–1.4 µM
Vorinostat + Pinometostat	SeAx	1.1	1.0–1.3 µM

## Discussion

Here, we used HDAC inhibitors and DOT1L inhibitors to study the potential crosstalk between HDACs and DOT1L in the context of CTCL treatment with clinically relevant pan-HDAC inhibitors. We have previously shown that HDAC1 is an evolutionary conserved negative regulator of DOT1L activity in yeast and mouse thymocytes (Vlaming et al., 2019). Mechanistically, in yeast Rpd3 activity is required to keep histone acetylation low and thereby suppress transcription, H2B ubiquitination, and Dot1 activity (Vlaming et al., 2019). This idea is further supported by the recent observation that histone H4 tail acetylation enhances yeast Dot1 activity *via* direct interactions on the nucleosome (Cutler et al., 2021; Valencia-Sánchez et al., 2021). Our previous results showed that HDAC1-DOT1L crosstalk is also relevant in the context of mouse thymocytes and thymic lymphoma (Vlaming et al., 2019). In this current study we looked into a more clinically relevant setting. HDAC inhibitors are already used in the clinic for treatment of CTCL (Mann et al., 2007; Jones et al., 2016; Wilcox, 2016; Lopez et al., 2018; Sermer et al., 2019; Chen et al., 2020; Lai and Wang, 2021b; Pavlidis et al., 2021). The precise mechanism of action remains to be discovered, however (Lopez et al., 2018; Chen et al., 2020). Here we investigated whether the previously identified crosstalk between HDAC and DOT1L occurs in human CTCL lines, addressing the question whether the crosstalk between DOT1L and HDAC1 observed in mouse T cells mediates the therapeutic effect of pan-HDAC inhibition in human CTCL.

Treatment of CTCL cells with the DOT1L inhibitor led to a progressive loss of H3K79me1 and H3K79me2. This time-dependent effect is in agreement with the notion that in many cell types there is no active demethylase for H3K79me. Instead, methylation of H3K79 by DOT1L is counteracted by histone turnover by replication-independent histone exchange or as a result of dilution due to cell replication (De Vos et al., 2011; Chory et al., 2019; Kwesi-Maliepaard et al., 2020). As a consequence, loss of H3K79 methylation upon inhibition of DOT1L is dependent on successive rounds of cell division. Gaining H3K79 methylation by increased DOT1L activity should not be dependent on histone turnover and dilution and can in principle occur on shorter time scales. Of note, Figures 2C, D show reduced cell viability of the CTCL cell lines upon treatment with high doses of the DOT1L inhibitors Pinometostat and SGC-0946. Since H3K79me is already strongly reduced at much lower concentrations of Pinometostat, and since the high doses were also toxic for DOT1L-inihibitor insensitive Jurkat cells (Daigle et al., 2011) (Supplementary Figure S2), the reduced viability of the CTCL cells was most likely mediated by off-target effects unrelated to loss of H3K79 methylation. In agreement with this, a recent study by Richter et al., observed that high doses of DOT1L inhibitors can cause effects on gene expression that are not the direct effect of loss of H3K79 methylation (Richter et al., 2021).

The HDAC inhibitor Vorinostat increased histone acetylation in the two CTCL lines, already after a short period of treatment. This is in line with other publications (Tiffon et al., 2011). Based on total H3K79 methylation levels we did not observe crosstalk between HDAC and DOT1L in the context of CTCL cells treated with the pan-HDAC inhibitor Vorinostat, i.e. treatment of CTCL cells did not lead to an increase in H3K79me or H3K79me2. In addition to crosstalk at the level of histone modification, we also independently investigated crosstalk at the functional level. Using viability assays with two human CTCL cell lines, we did not see antagonism or synergy between HDAC and DOT1L inhibition.

However, this does not necessarily exclude the presence of crosstalk between DOT1L and HDAC1 in CTCL, as there are several differences between the conditions used here and those of the previous study of Vlaming et al. (Vlaming et al., 2019). Vlaming et al. used complete and permanent deletion of one HDAC (HDAC1) in mouse cells while here we used short-term partial inhibition of multiple HDACs in human cells. It is possible that the remaining HDAC activity in the study here was sufficient to ensure normal DOT1L activity. Alternatively, the inhibition of multiple HDACs by the pan-HDAC inhibitors used here may have suppressed the effect of loss of function of HDAC1 alone. To investigate this possibility, more specific inhibitors could be used in future studies. Class-I specific HDAC inhibitors are available for such studies, but inhibiting HDAC1 or other specific class-I HDACs remains a challenge (Schölz et al., 2015; Eckschlager et al., 2017; Shortt et al., 2017; Pride and Summers, 2018; Hess et al., 2022; Siklos and Kubicek, 2022). Interestingly, recent efforts have led to the development of more selective HDAC inhibitors, some of which inhibit certain class-I HDACs more than others (Yang et al., 2019; Hess et al., 2022; Siklos and Kubicek, 2022).

Another major difference is that loss of HDAC1 promotes tumorigenesis in mouse thymocytes, whereas in human CTCL loss of HDAC activity has anti-tumor effects. This study focused on two commonly used CTCL cell lines, for which we did not observe sensitivity towards DOT1L inhibition. However, the observation of fusion proteins involving DOT1L in mycosis fungoides suggests that there might be specific CTCL subtypes that are sensitive to DOT1L inhibitors. To our knowledge, DOT1L sensitive CTCL subtypes have not been reported so far.

It is also possible that there is crosstalk between HDAC and DOT1L on a different level that does not directly impact cell viability. HDACs have multiple substrates beside histones (Singh et al., 2010; Drazic et al., 2016), indicating that part off the effect of HDAC inhibitors might be the result of increased acetylation of other proteins in the cell. For DOT1L there has only been one other substrate reported besides H3K79, the androgen receptor (AR) (Yang et al., 2013). It is thus possible that HDAC1 and DOT1L show crosstalk on the level of posttranslational modifications of other putative, yet unknown substrates. However, since DOT1L inhibition did not modify the sensitivity to HDAC inhibition, these putative alternative levels of crosstalk are not likely involved in the mechanisms of action of HDAC inhibitors.

Pan-HDAC inhibitors have pleiotropic effects in CTCL and exact mechanism(s) by which they affect cell viability of CTCL cells is not completely known, although it has been shown that combined treatment of an HDAC inhibitor with a demethylating agent led to re-expression of a tumor suppressor gene and induction of apoptosis in CTCL cell lines (Duvic, 2015; Rozati et al., 2016; Moskowitz and Horwitz, 2017; Lopez et al., 2018; Chen et al., 2020). HDAC inhibitors are used in a variety of cancer types, often when HDAC activity is increased (West and Johnstone, 2014). However, in CTCL only expression of HDAC9 is increased (Lee et al., 2012; Qu et al., 2017). It has been suggested that during treatment of CTCL patients with HDAC inhibitors the CTCL cells are not the only responders to treatment. The non-malignant CD4^+^ T cells from the host also respond to HDAC inhibitor treatment and might be equally important for the overall response (Ott and Wu, 2017; Qu et al., 2017). In this current study we did not address the effect of both inhibitors on non-malignant T cells, but other studies have looked at the effect of single HDAC- or DOT1L-inhibitors in T cells. Inhibition of DOT1L during *in vitro* mouse T cell culture alleviates the T cell receptor stimulation threshold, thereby attenuating graft-versus-host-disease in allogeneic T cell transplantation (Kagoya et al., 2018). Furthermore, DOT1L is essential for normal CD4^+^ and CD8^+^ T cell development and differentiation in mice (Bian et al., 2020; Kwesi-Maliepaard et al., 2020; Scheer et al., 2020; Wille and Sridharan, 2022). The role of HDACs in CD4^+^ T cells has been studied extensively. HDACs play important roles in thymic CD4^+^ T cell development and peripheral CD4^+^ T cell functionality and differentiation (Ellmeier and Seiser, 2018). Inhibition of HDACs in human CD4^+^ T cells induces regulatory T cell differentiation and affects cytokine expression (Ellmeier and Seiser, 2018). Given the important roles of HDAC and DOT1L in lymphocytes (Haery et al., 2015; Ellmeier and Seiser, 2018; Kealy et al., 2020; Kwesi-Maliepaard et al., 2020; Scheer et al., 2020; Aslam et al., 2021; Wille and Sridharan, 2022) it would certainly be interesting the assess the effect of HDAC inhibitors in combination with DOT1L inhibitors on normal immune cells and in other types of lymphoma. Taken together, although we could not find synergistic or suppressive effects of HDAC- and DOT1L-inhibitors in CTCL during short term *in vitro* cell culture, the crosstalk between HDAC and DOT1L still warrants further investigation, especially in the context of *in vivo* immune responses.

## Data Availability

The original contributions presented in the study are included in the article/Supplementary Material, further inquiries can be directed to the corresponding author.

## Abbreviations

CTCL, Cutaneous T-cell lymphoma; DOT1L, DOT1-like histone lysine methyltransferase; H3K79me, methylation of lysine 79 on histone H3; H2BK120ub1, monoubiquitination of lysine 120 of histone H2B; HDAC, histone deacetylase; KDM6A, lysine demethylase 6A; MF, mycosis fungoides; MLL, Mixed-Lineage Leukemia; SS, Sézary syndrome; EC_50_, Half maximal effective concentration.
