# Supplementary material for: DOT1L inhibition does not modify the sensitivity of cutaneous T cell lymphoma to pan-HDAC inhibitors in vitro
Source: Front Genet. 2022 Nov 8;13:1032958. doi: 10.3389/fgene.2022.1032958 (PMC9681147; doi:10.3389/fgene.2022.1032958)
Supplement: Supplementary file 3 [file Presentation1.pdf]

Supplementary material

DOT1L inhibition does not modify the sensitivity of cutaneous T cell lymphoma to pan-HDAC inhibitors *in vitro*

Eliza Mari Kwesi-Maliepaard[1,*], Muddassir Malik[1,*], Tibor van Welsem[1], Remco van Doorn[2], Maarten H. Vermeer[2], Hanneke Vlaming[1], Heinz Jacobs[3] and Fred van Leeuwen[1,4]

[1] Division of Gene Regulation, Netherlands Cancer Institute, 1066CX Amsterdam, The Netherlands

[2] Department of Dermatology, Leiden University Medical Center, 2300 RC Leiden, The Netherlands

[3] Division of Tumor Biology and Immunology, Netherlands Cancer Institute, 1066CX Amsterdam, The Netherlands

[4] Department of Medical Biology, Amsterdam UMC, University of Amsterdam, 1105AZ Amsterdam, The Netherlands

* These authors contributed equally

Corresponding author: Fred van Leeuwen, fred.v.leeuwen@nki.nl

Additional files

Additional_file1.xlsx: Raw data for cell viability curves

Additional_file2.xlsx: Raw data for Figure 2E and 2F

Additional_file3.xlsx: Raw data for Figure 2G and 2H

Supplementary Figure S1

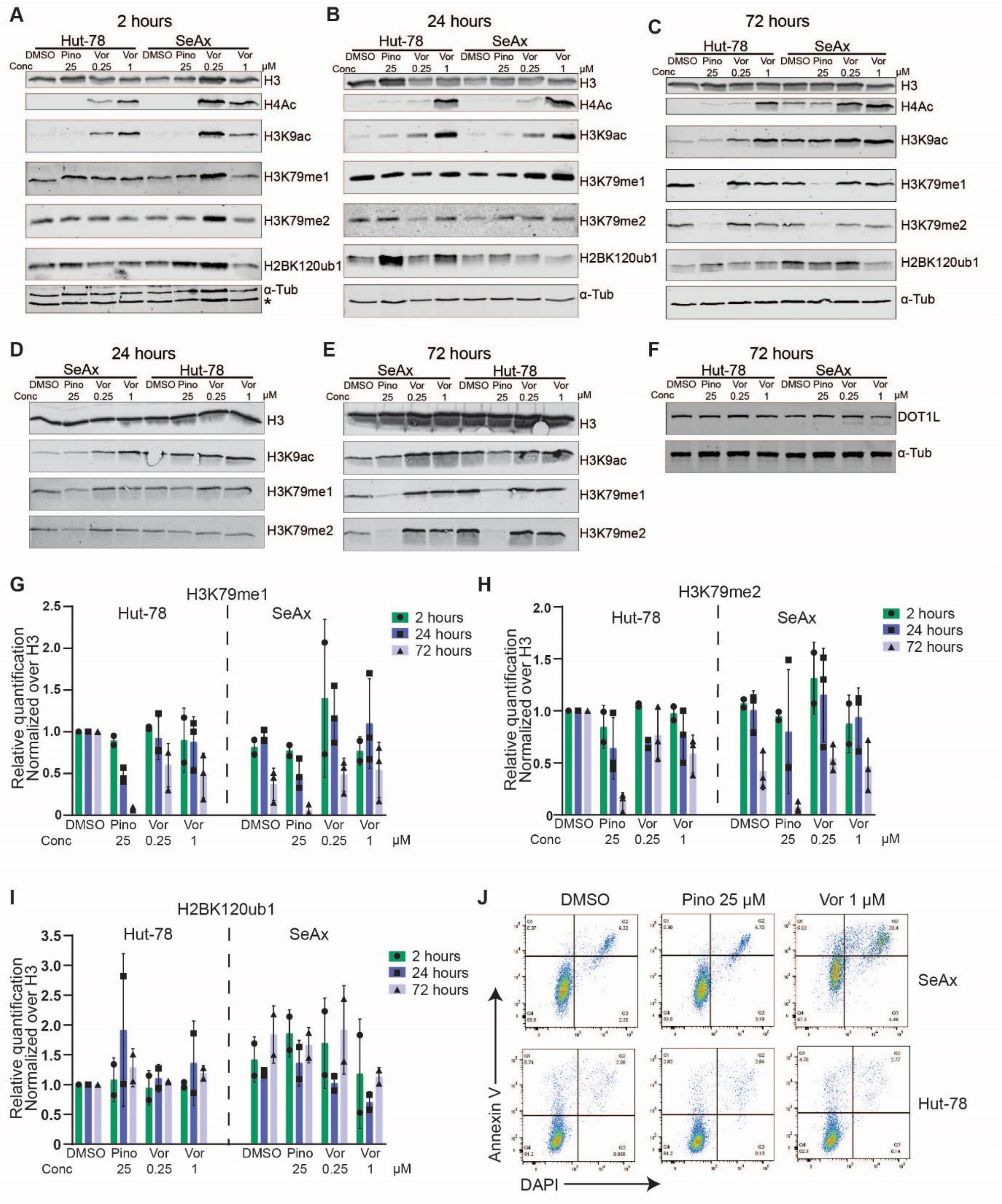

Figure S1: Histone modification levels after treatment of CTCL lines with HDAC inhibitors and DOT1L inhibitors. A-C) Western blots showing H3K79me1/2, pan H4ac, H3K9ac and H2BK120ub1 levels in the indicated CTCL cell lines after treatment with 25 µM Pinometostat (Pino), and 0.25 µM or 1 µM Vorinostat (Vor) for 2 hours (A), 24 hours (B) or 72 hours (C). D) Western blot on unsonicated samples showing DOT1L protein levels in the indicated CTCL cell lines after treatment with 25 µM Pinometostat (Pino), 0.25 µM or 1 µM Vorinostat for 72 hours. E-F) Cell viability of the indicated CTCL cell lines after treatment with 25 µM Pinometostat (Pino), and 0.25 µM or 1 µM Vorinostat for 72 hours determined by Annexin V-DAPI staining. Bars indicate average value of two independent biological replicates; individual data points are shown.

Supplementary Figure S2

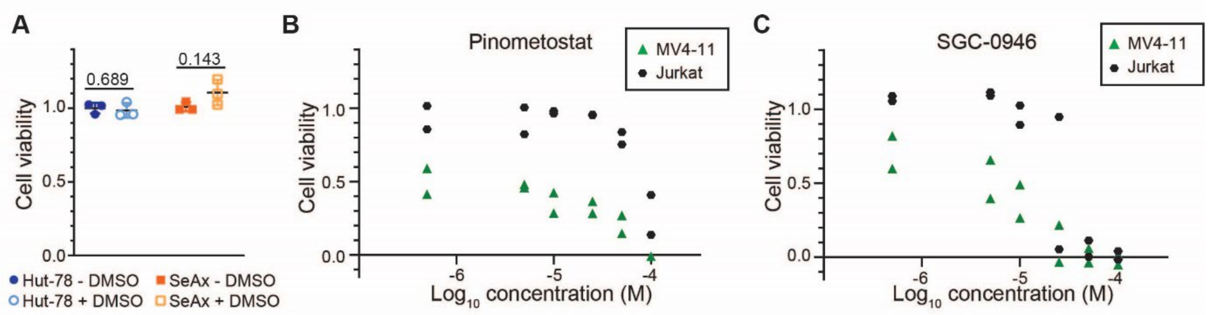

Figure S2: CTCL cell viability upon treatment with HDAC inhibitors and/or DOT1L inhibitors. A-D) Cell viability of the CTCL cell lines Hut-78 and SeAx after 72 hours of treatment with HDAC inhibitors Vorinostat (A) and Panobinostat (B) and DOT1L inhibitors Pinometostat (C) and SGC-0946 (D). Points show average values of three independent biological replicates ± SD. E-F) Cell viability of Hut-78 cells (E) and SeAx cells (F) treated with increasing concentrations of Pinometostat and with 2 µM Vorinostat (Hut-78) or 1 µM Vorinostat (SeAx) or without Vorinostat. Bar plots show average values of three independent biological replicates ± SD and individual data points. G-H) Cell viability of Hut-78 (G) and SeAx (H) cells treated with increasing concentrations of Vorinostat and with or without 25 µM Pinometostat. Bar plots show average values of three independent biological replicates ± SD and individual data points. *P*-values derived from unpaired student's t-test are indicated. Cell viability was determined using a Cell Titer Blue assay and normalized against untreated cells. Each biological replicate represents the average of three technical replicates.
